# Baicalin enhances respiratory mucosal immunity by modulating antiviral protein expression and T-cell homeostasis during H9N2 infection

**DOI:** 10.3389/fimmu.2025.1593691

**Published:** 2025-05-08

**Authors:** Zhenyi Liu, Haotong Guo, Yan Zhi, Xiaorui Jiang, Qian Zhang

**Affiliations:** ^1^ College of Animal Science and Technology, Beijing University of Agriculture, Beijing, China; ^2^ YanTai Yuhuangding Hospital Affiliated to Qingdao University, Yantai, China

**Keywords:** baicalin, H9N2 avnia influenza virus, respiratory mucosal immunity, antiviral proteins, MX1, PKR, natural immunomodulators

## Abstract

**Introduction:**

The H9N2 avian influenza virus, although not highly pathogenic, still poses ongoing risks to poultry health and food security due to its ability to resist vaccines and its potential to spread to humans.

**Methods:**

This study investigated the effects of baicalin, a flavonoid derived from *Scutellaria baicalensis*, on respiratory mucosal immunity during H9N2 infection. In vitro experiments were conducted using MLE-12 alveolar epithelial cells, and in vivo evaluations were performed in a mouse model of H9N2 infection.

**Results:**

Baicalin treatment enhanced the expression of antiviral proteins Mx1 and PKR in a dose- and time-dependent manner, helping to counteract the virus’s suppression of these defense proteins. In addition to strengthening this epithelial barrier, baicalin has both antiviral and immune-regulating effects: it directly blocks viral replication and helps restore the CD4+/CD8+ T cell ratio in H9N2-infected mice. Most importantly, baicalin reduces lung damage and spleen shrinkage while keeping the immune system balanced. These results show that baicalin enhances mucosal antiviral defenses by simultaneously regulating innate antiviral pathways (Mx1 and PKR) and restoring adaptive immune balance (CD4+/CD8+ T-cell ratio).

**Discussion:**

These dual protective effects highlight baicalin’s potential as a natural therapeutic strategy for improving mucosal immunity against vaccine-resistant influenza viruses such as H9N2, contributing valuable insights into plant-derived immunomodulatory approaches against emerging zoonotic viral threats.

## Graphical Abstract

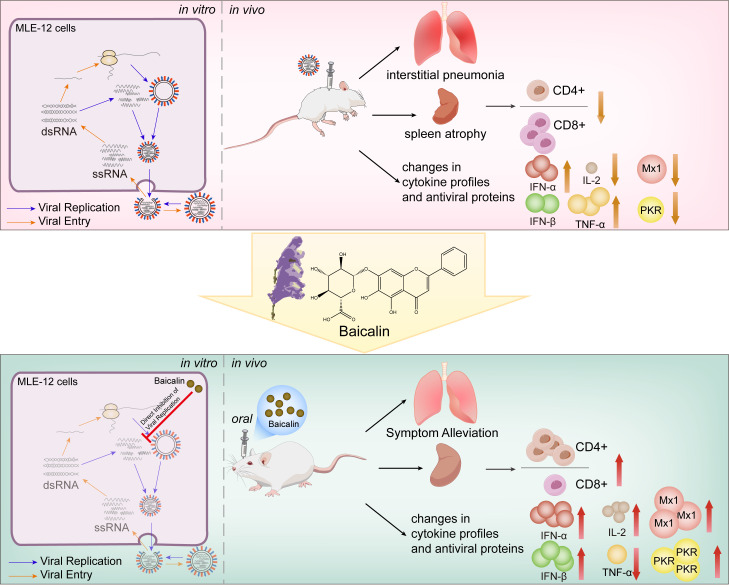

## Introduction

Avian influenza viruses (AIV), especially the H9N2 subtype, represent an enduring challenge for global animal and human health, characterized by persistent prevalence, frequent genetic reassortment, and potential for zoonotic transmission (1, 2). Despite its classification as a low pathogenic virus, H9N2’s extensive dissemination and adaptability pose significant economic threats to poultry production and substantial public health risks (3, 4). Vaccination remains a critical control strategy; however, rapid antigenic drift and vaccine-induced immune escape severely compromise long-term efficacy (5, 6). Furthermore, H9N2 infection predominantly targets the respiratory mucosal barrier, disrupting alveolar epithelial integrity and evading initial immune surveillance mechanisms (7). Consequently, this vulnerability highlights the urgent need for therapeutic strategies that specifically enhance mucosal immunity and strengthen coordination between local mucosal defenses and systemic immune responses (8).

Mucosal barriers in respiratory and gastrointestinal tracts function as primary defense interfaces against pathogen invasion, orchestrating immune responses critical to pathogen clearance and host homeostasis (9–11). Alveolar epithelial cells, key sentinels of respiratory mucosa, produce antiviral proteins such as interferons (IFNs) and immune-regulatory cytokines, bridging local mucosal defense with systemic immune activation (12, 13). However, current knowledge regarding how natural compounds modulate these intricate mucosal-systemic immune interactions during respiratory viral infections remains insufficiently understood, particularly for influenza viruses, presenting a critical research gap.

Baicalin, a bioactive flavonoid glycoside extracted from the medicinal herb Scutellaria baicalensis, has shown considerable therapeutic promise due to its broad-spectrum antiviral and immunomodulatory properties (14, 15). Previous studies have demonstrated baicalin’s capability to inhibit viral neuraminidase activity in H1N1 and H3N2 infections (16) and suppress inflammation induced by H7N9 through regulation of caspase-mediated cell death (17). However, these studies largely focused on systemic antiviral responses and lacked detailed exploration of mucosal immunity, as well as *in vivo* validation specifically for H9N2 AIV infection. Importantly, baicalin’s pharmacokinetic profile favors mucosal retention, which significantly enhances intestinal mucosal barrier function and immune regulation (18, 19). Nevertheless, its potential role in modulating respiratory mucosal barrier immunity, particularly in H9N2 AIV infections, remains largely unexplored. Specifically, the integration between baicalin’s direct antiviral effects and its modulation of immune signaling pathways (e.g., TLRs/NF-κB, PI3K/AKT, JAK-STAT) at the respiratory mucosal interface warrants comprehensive investigation (20, 21).

Therefore, we hypothesized that baicalin confers antiviral protection primarily through interferon-mediated induction of antiviral proteins (Mx1 and PKR) at the respiratory mucosal barrier during H9N2 AIV infection. Additionally, we explored whether baicalin simultaneously supports the restoration of CD4+/CD8+ T-cell homeostasis and modulation of virus-induced apoptosis as secondary mechanisms to reinforce systemic immune responses and mucosal epithelial integrity (22). To test this hypothesis, we employed an integrative experimental approach combining *in vitro* studies with mouse alveolar type II epithelial cells (MLE-12) and an *in vivo* murine infection model. Through this design, we comprehensively evaluated baicalin’s effects on mucosal immune signaling, antiviral protein expression, cytokine dynamics, and systemic immune integration following H9N2 infection. The outcomes of this study offer novel insights into how baicalin modulates mucosal and systemic immune interactions, enhancing respiratory mucosal barrier defense. By elucidating baicalin’s detailed antiviral and immunomodulatory mechanisms, this research contributes to the broader understanding of mucosal immunity modulation by natural compounds and supports the development of innovative therapeutic strategies for respiratory pathogens, particularly those resistant to conventional vaccine approaches such as H9N2 AIV.

## Materials and methods

### Ethics approval and consent to participate

The animal research protocols for this study were designed in strict accordance with the *International Guiding Principles for Biomedical Research Involving Animals*, ensuring adherence to the highest standards of animal welfare (https://grants.nih.gov/grants/olaw/Guiding_Principles_2012.pdf). Ethical approval was obtained from the Animal Care and Use Committee of the Institute of Animal Husbandry and Veterinary Medicine (Permit number: BUA20240723). In line with these guidelines, comprehensive measures were implemented to minimize animal suffering and enhance welfare. These measures included the use of anesthetics, optimized handling techniques, and adherence to the 3Rs (replacement, reduction, and refinement) to minimize the impact on the animals involved (23).

### Cell lines and viruses

Madin–Darby Canine kidney (MDCK) cells and mouse alveolar type II epithelial cells (MLE-12; American Type Culture Collection, CRL-2110) were used as cellular models. MDCK cells were cultured in Dulbecco’s Modified Eagle’s Medium (DMEM; BioWhittaker, Walkersville, MD, USA) supplemented with 10% fetal bovine serum (FBS; Gibco-BRL, New York, NY, USA) and 2% penicillin-streptomycin (Gibco-BRL). MLE-12 cells were maintained similarly in DMEM supplemented with 10% FBS and antibiotics. Both cell lines were incubated under standard culture conditions (37°C, 5% CO_2_, humidified atmosphere).

The H9N2 AIV subtype (GenBank accession number FJ499463) used in this study was provided by the China Agricultural University laboratory. Viral propagation was performed in MDCK and MLE-12 cells and further amplified in 11-day-old specific pathogen-free (SPF) embryonated chicken eggs according to established protocols (24). Viruses were stored at -80°C until use, and titers were routinely determined by plaque assays.

### Baicalin characterization and cytotoxicity assessment

Baicalin, a bioactive flavonoid monomer derived from *Scutellaria baicalensis* known for its antiviral and immunomodulatory properties (25, 26), was purchased from Shanghai Tongtian Biotechnology Co., Ltd. For cytotoxicity assessment, MLE-12 cells were seeded in 96-well polypropylene plates at a density of 1 × 10^4^ cells/mL, with 100 µL of medium per well. The cells were incubated at 37°C in a 5% CO_2_ atmosphere to ensure optimal cell adherence and growth. A broad range of Baicalin concentrations (0–100 µg/mL) was tested using a serial dilution approach to determine the optimal concentration. MLE-12 cells were exposed to Baicalin concentrations of 2.5, 5, 10, 25, 50, and 100 µg/mL. Cell viability was assessed using the protocol described above, and the optimal concentration range was chosen based on maintaining ≥80% cell viability. Upon reaching 80% confluence, cells were treated with varying concentrations of Baicalin (2.5, 5, and 10 µg/mL), dissolved in phenol-red free DMEM with dimethyl sulfoxide (DMSO) as the solvent, ensuring a non-toxic solvent concentration across all treatments. Each concentration was tested in six replicates, with control wells receiving DMEM supplemented only with DMSO. Peripheral wells were filled with phosphate-buffered saline (PBS) (pH 7.2-7.4) to prevent edge evaporation. Cell viability was assessed at 12, 24, 36, and 48 hours post-treatment by rinsing the wells with 200 µL PBS, followed by the addition of 10 µL CCK-8 reagent (Beijing Solar-bio Science & Technology Co., Ltd.). After 2–4 hours of incubation to allow for formazan crystal development, absorbance was measured at 450 nm (formazan detection wavelength) with a reference wavelength of 650 nm for background subtraction, according to the CCK-8 manufacturer’s protocol. Cell viability was calculated using the following formula:


$$\text{Cell Viability }\left(\%\right)=\frac{\text{Absorbance of Treatment}}{\text{Absorbance of Control​}}\times 100\%$$

### Viral titer determination and multiplicity of infection calculation in H9N2 AIV infection

To assess the replicative capacity of the H9N2 AIV, viral titration was performed using MDCK cells. After removing the growth medium, the cells were washed three times with pre-warmed D-Hank’s solution to eliminate any residual serum. The virus was serially diluted in 10-fold increments and added to the cells in triplicate, along with a virus-free control for experimental validation. Control wells received DMEM alone to ensure assay validity. Virus adsorption was facilitated by incubating plates at 37°C for 1 hour with gentle agitation every 20 minutes. After removing excess virus, cells were washed with DMEM to remove unbound viral particles. Subsequently, cell monolayers were overlaid with 2% low-melting point agarose solution mixed with equal volumes of 2× DMEM. The agarose overlay was briefly solidified at 4°C, followed by incubation at 37°C in a 5% CO_2_ humidified incubator for 72 hours to allow plaque development. After fixation in 10% formaldehyde and staining with 1% crystal violet, plaques were counted and viral titers (plaque-forming units [PFU]/mL) were determined using the following formula:


$$\text{PFU}/\text{mL}=\frac{\text{Average Plaques }\left(\text{n}\right)\times \text{Dilution Factor​​}}{\text{Virus Inoculum​}}$$

The optimal MOI for infecting MLE-12 cells was subsequently determined. MLE-12 cells seeded in six-well plates were infected with H9N2 AIV at varying MOIs (0, 0.01, 0.05, and 0.1). A 1-hour adsorption period at 37°C in a 5% CO_2_ incubator was used to facilitate viral entry. Following adsorption, unbound virus particles were removed by washing cells twice with DMEM. Cells were then cultured under standard conditions, and supernatants were harvested at predetermined intervals (12, 24, 36, and 48 hours post-infection) from triplicate wells for viral titration by plaque assay.

### Experimental design and grouping for *in vitro* and *in vivo* studies with baicalin

#### *In vitro* studies

MLE-12 cells were cultured in six-well plates and divided into five experimental groups: a blank control (no virus or treatment), a virus control (virus infection without treatment), and three baicalin-treated groups at concentrations of 5 (low), 10 (medium), and 15 µg/mL (high). These dosages were selected based on the cytotoxicity results obtained from our preliminary cell viability assays and are consistent with concentrations previously reported as effective yet minimally toxic in antiviral and immunomodulatory *in vitro* studies (27). Each group was performed in triplicate. Upon reaching approximately 90% confluence, cells were infected with H9N2 AIV at an MOI of 0.05 in serum-free DMEM; the blank control received only serum-free DMEM. After a 1-hour viral adsorption period at 37°C, unbound virus was removed by washing the cells twice with DMEM. Supernatants were collected at intervals of 12, 24, 36, and 48 hours for subsequent analysis of viral replication and antiviral activity.

The inhibition rate was determined using the following formula:


$$\begin{array}{c} \text{Inhibition Rate }\left(\%\right)\\ =\frac{\text{Virus Group Lung Index }-\text{ Treatment Group Lung Index​​}}{\text{Virus Group Lung Index​}}\times 100\% \end{array}$$

#### *In vivo* studies

Sixty SPF mice (6–8 weeks old, weighing 15–18 grams) were randomly divided into six groups, with an equal number of male and female mice. The mice were sourced from the Xinglong Experimental Animal Breeding Farm (Beijing). Experimental groups included three baicalin-treated groups (low, medium, and high dosages), one positive control group treated with ribavirin, one virus-infected model group receiving no treatment, and one blank control group not infected or treated. Baicalin dosages (80, 160, and 320 mg/kg/day, corresponding to low, medium, and high doses, respectively) were calculated based on human clinical equivalent dosages using a standard interspecies dosage conversion formula recommended by the U.S. Food and Drug Administration (FDA) (https://www.fda.gov/media/72309/download), accounting for differences in body surface area between humans and mice. Ribavirin was administered at a clinically relevant dose of 10 mg/kg/day as a positive antiviral control (28). H9N2 AIV infection was established intranasally in all experimental groups, except the blank control. Baicalin and ribavirin treatments were initiated immediately post-infection, administered orally twice daily at a total volume of 0.8 mL/mouse/day (0.4 mL per dose). The virus-infected model group received an equivalent volume of DMEM without drugs, and the blank control was neither infected nor treated. Throughout the treatment period, mice were provided free access to food and water, with health status and behavioral parameters monitored closely. At 48 hours post-infection, all mice were humanely euthanized, and samples of lung, spleen, and blood (via ocular vein) were collected for subsequent analysis to evaluate antiviral and immunomodulatory effects compared to ribavirin.

### Histopathological observations

Following the infection and treatment period, lung tissues from the mice were harvested for histopathological evaluation. The tissues were rinsed with PBS and fixed in 10% formalin for 24 hours to preserve cellular integrity. After fixation, the tissues were processed for histological analysis, embedded in paraffin, and sectioned into 4 µm-thick slices. Hematoxylin and eosin (H&E) staining was applied to evaluate tissue morphology. Histopathological analyses were conducted using a DP80 Digital Light Microscope (Olympus, Tokyo, Japan). Lung injury severity was quantitatively assessed using Image-Pro Plus 6.0 software.

The degree of lung pathology was classified as previously described with minor modifications (29, 30): mild (presence of exudates in some alveoli, a small number of inflammatory cells around bronchioles, and mild capillary dilation and congestion), moderate (partial consolidation of lung tissue, extensive inflammatory infiltration around bronchioles, and exudates in alveoli), or severe (widespread consolidation of lung tissue, extensive infiltration of mononuclear cells, and significant obstruction of bronchioles and alveoli due to inflammatory exudates). Each lung tissue sample was examined across at least 10 fields to ensure the accuracy and robustness of the findings. Additionally, lung indices were calculated to quantitatively evaluate the extent of lung involvement and assess the therapeutic efficacy of baicalin treatment.

### RNA extraction and real-time quantitative PCR

Total RNA was extracted from MLE-12 cells using TransZol Up reagent (Takara, Dalian, China) according to the manufacturer’s instructions. Extracted RNA was reverse transcribed into complementary DNA (cDNA) using a high-fidelity reverse transcription kit, and the resulting cDNA was stored at −80°C for subsequent analysis. RT-qPCR was performed using the 2× Hieff UNICON^®^ Universal Blue qPCR Master Mix (Yeasen, Shanghai, China). The amplification protocol consisted of UDG activation at 50°C for 2 minutes, initial denaturation at 95°C for 2 minutes, followed by 40 cycles of amplification of 95°C for 15 seconds and 60°C for 30 seconds. A melting curve analysis was conducted following amplification to verify the specificity of the PCR products. Each sample was analyzed in triplicate to ensure reproducibility. GAPDH was used as the internal reference gene, as described by Meng et al. (31). Gene expression fold changes were calculated using the 2^−ΔΔCT^ method, with the results normalized to the expression of GAPDH expression. Primer sequences for all target genes are listed in **Table 1**. RT-qPCR data analysis and statistical comparisons were performed using GraphPad Prism 8.0 software (GraphPad Software Inc., San Diego, CA, USA).

**Table 1:** Primers used for real-time quantitative PCR.

Genes	Sequence (5’-3’)	Product Length (bp)	Gene ID
**IFN-α**	F: GCGTTCCTGCTGTGCTTCT R: TCTGCATCTTCTCCGTCATCTC	178 bp	111654
**IFN-β**	F: GCTACTGGCCAACCTGCTCT R: GGCTTGAGCCTTCTTGATCTG	189 bp	15977
**Mx1**	F: GCAACCAGCCTGCAGACATA R: GCTCAGAGCCTCTGTGGTAGC	128 bp	17857
**PKR**	F: ACCTTCAGTTTGGGCAAGATG R: CCTTCACCTGTGCAAGTTATGTC	185 bp	23992
**GAPDH**	F: GTCCTCAGTGTAGCCCAAGAT R: CAATGTGTCCGTCGTGGATCT	125 bp	14433

### Enzyme-linked immunosorbent assay determination of cytokine levels

Cytokine concentrations in both *in vitro* and *in vivo* samples were quantified using ELISA. For serum collection in the *in vivo* experiments, blood samples were drawn via the ocular vein of mice, allowed to coagulate at room temperature for 30 min, and further refrigerated overnight at 4°C. Subsequently, serum was obtained by centrifugation at 2,000 rpm for 15 min at 4°C, and the supernatant was stored at −80°C until analysis. *In vitro* analyses focused on measuring type I interferons (IFN-α and IFN-β) in the culture supernatants of MLE-12 cells, whereas *in vivo* assays evaluated interleukin-2 (IL-2) as an indicator of T-cell activation and tumor necrosis factor-alpha (TNF-α) to assess systemic inflammatory responses in mice infected with H9N2 AIV. Lung tissue samples were homogenized in pre-cooled PBS containing protease inhibitors, followed by centrifugation at 1,000 rpm for 10 min at 4°C to collect supernatants for cytokine analysis. ELISA assays were performed using commercial kits (ABclonal Biotechnology, Wuhan, China) according to the manufacturer’s protocols. Optical density (OD) measurements were obtained at 450 nm wavelength using a TECAN F50 spectrophotometer (TECAN Group Ltd., Männedorf, Switzerland). Cytokine concentrations in samples were calculated using standard curves generated from known concentrations of cytokine standards provided by the kit.

### Western blotting analysis for protein expression in MLE-12 cells and lung tissue

Protein expression changes in MLE-12 cells and mouse lung tissues were assessed by western blotting. Samples were stored at −80°C until analysis. Cells and tissues were homogenized in ice-cold RIPA buffer supplemented with phenylmethylsulfonyl fluoride (PMSF, protease inhibitor) at a ratio of 100:1 (v/v). Homogenates were centrifuged at 12,000 rpm for 20 min at 4°C, and the protein-containing supernatants were collected. Protein concentrations were determined using a BCA assay kit (Beyotime, Shanghai, China), following the manufacturer’s instructions.

Proteins were separated by 12% SDS-PAGE and transferred onto polyvinylidene fluoride (PVDF) membranes. After transfer, the membranes were blocked with 5% non-fat milk in PBST and incubated overnight at 4°C with primary antibodies targeting Mx1 and PKR (Jiangsu Qinke Biotechnology), while GAPDH (Wuhan Sanying Biotechnology) was used as the internal control. After washing with TBST three times (10 min each), the membranes were incubated with appropriate secondary antibodies for 1 hour at room temperature. Protein bands were visualized using an Odyssey Infrared Imaging System (LI-COR Biosciences, Lincoln, NE, USA). Protein expression levels were quantified using ImageJ software (National Institutes of Health, Bethesda, MD, USA), and relative expression values were normalized to GAPDH to account for variations in protein loading.

### Evaluation of the effects of baicalin on T lymphocyte subpopulations in H9N2-infected mice

To investigate the immunomodulatory effects of baicalin on T lymphocyte subpopulations following H9N2 infection, spleen tissues from treated mice were harvested and analyzed by flow cytometry. Spleen tissues were minced and ground to generate a single-cell suspension, followed by centrifugation at 300 ×g for 5 minutes to remove tissue debris. The resulting splenocytes were resuspended in PBS and stained for flow cytometric analysis using fluorochrome-conjugated antibodies specific for surface markers CD3, CD4, CD8, and CD49b. After staining, cells were washed and resuspended in PBS for flow cytometric analysis. Data were collected from 20–000 events per sample. The ratio of CD4+/CD8+ T cells was calculated and expressed as mean ± standard deviation for statistical comparison.

### Statistical analysis

Data analysis was performed using GraphPad Prism (Version 8.0, GraphPad Software Inc., San Diego, CA, USA). Results are presented as means ± standard deviations. Differences between two independent groups were evaluated using an unpaired Studen’s t-test, while comparisons among multiple groups were performed using one-way analysis of variance (ANOVA) followed by Tukey’s *post-hoc* test. Gene expression data from RT-qPCR were analyzed using the 2^−ΔΔCT^ method, and protein band intensities from western blotting were quantified with ImageJ (Version 1.53, National Institutes of Health, Bethesda, MD, USA). A *P*-value < 0.05 was considered statistically significant.

## Results

### Establishing the optimal MOI for H9N2 AIV in MLE-12 cells

**Figure 1:**
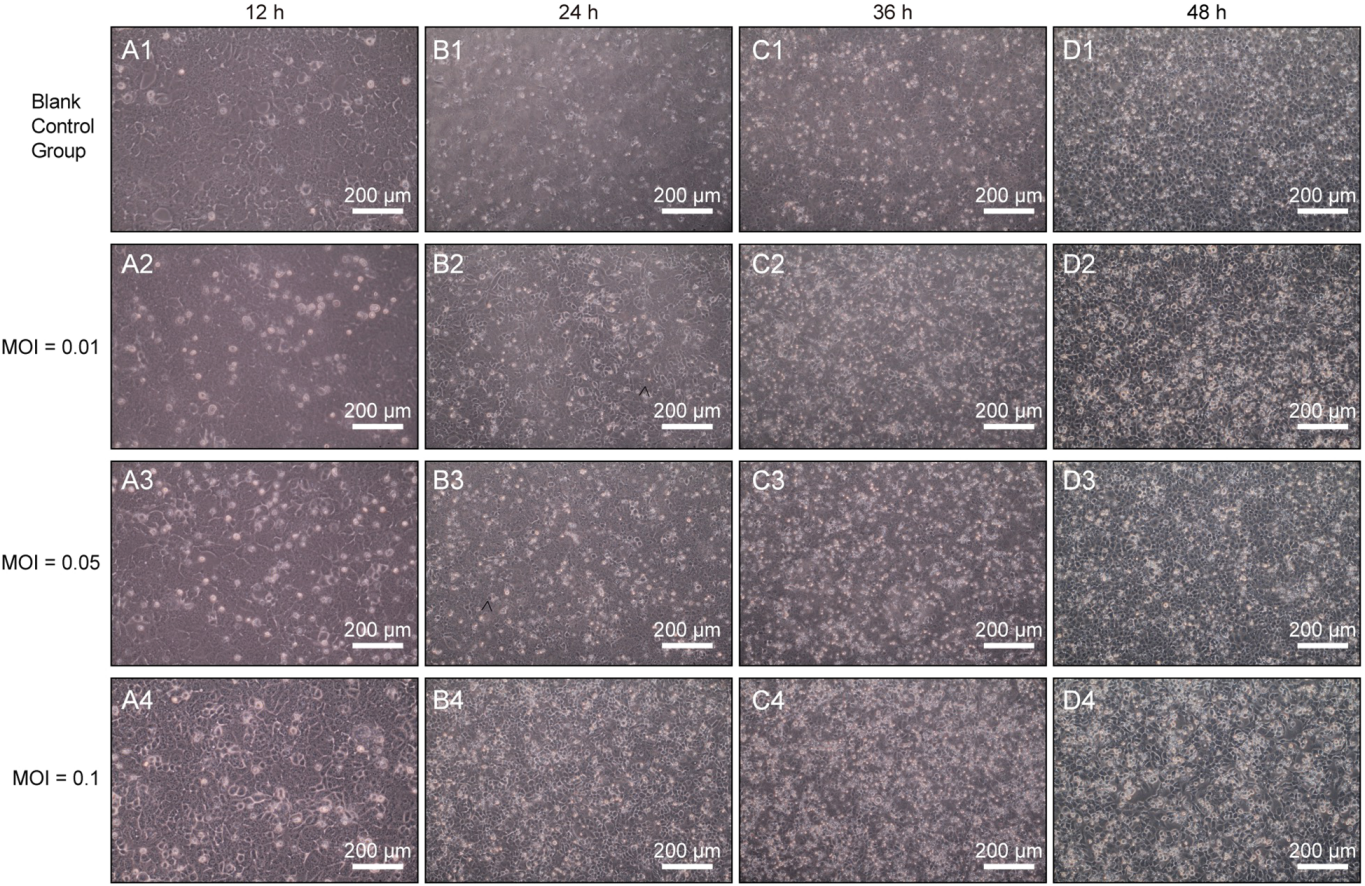
Morphological assessment of H9N2 AIV infection in MLE-12 cells at different MOIs (200×). Representative images of MLE-12 cells infected with H9N2 AIV at MOIs of 0.01, 0.05, and 0.1 were captured at 12 h **(A1–A4)**, 24 h **(B1–B4)**, 36 h **(C1–C4)**, and 48 hpi **(D1–D4)**. Scale bars: 200 µm.

To determine the optimal MOI for H9N2 AIV in MLE-12 cells, we investigated virus-cell interactions under different MOIs. At 12 hours post-infection (hpi), cellular swelling and mild cytopathic effects were initially observed at an MOI of 0.05 (**Figures 1, A3**), whereas no significant morphological changes occurred in the control group (**Figures 1, A1**). At 24 hpi, cells infected at MOI 0.05 exhibited pronounced morphological alterations, including cell rounding and focal disruptions of the monolayer (**Figures 1, B3**). At 36 hpi, cytopathic effects intensified, resulting in partial cell detachment and extensive disruption of the monolayer structure at MOIs of 0.05 and 0.1 (**Figures 1, C3–C4**). These effects were most severe by 48 hpi, particularly evident at MOI 0.1, characterized by widespread cellular detachment, extensive cell death, and complete loss of monolayer integrity (**Figures 1, D4**). Notably, cells infected at an MOI of 0.01 exhibited minimal cytopathic changes, suggesting limited virus replication under this condition.

Plaque assay analysis revealed distinct viral replication efficiencies among different MOIs. At an MOI of 0.05, PFU/mL were quantified as follows: 0.4 × 10³ PFU/mL at 12 hpi, 0.8 × 10³ PFU/mL at 24 hpi, 1.2 × 10³ PFU/mL at 36 hpi, and 2 × 10³ PFU/mL at 48 hpi. However, at an MOI of 0.1, severe and rapid cytopathic effects caused significant cell detachment and widespread loss of cell integrity, which impeded accurate plaque quantification (32). Therefore, an MOI of 0.05 supported optimal viral replication while maintaining sufficient monolayer integrity for further analysis. Consequently, based on both viral replication efficiency and maintenance of cell viability, an MOI of 0.05 with 1-hour virus adsorption at 37°C was selected as the optimal infection condition for subsequent experiments.

### Identifying optimal baicalin concentrations for antiviral assays in MLE-12 cells

**Figure 2:**
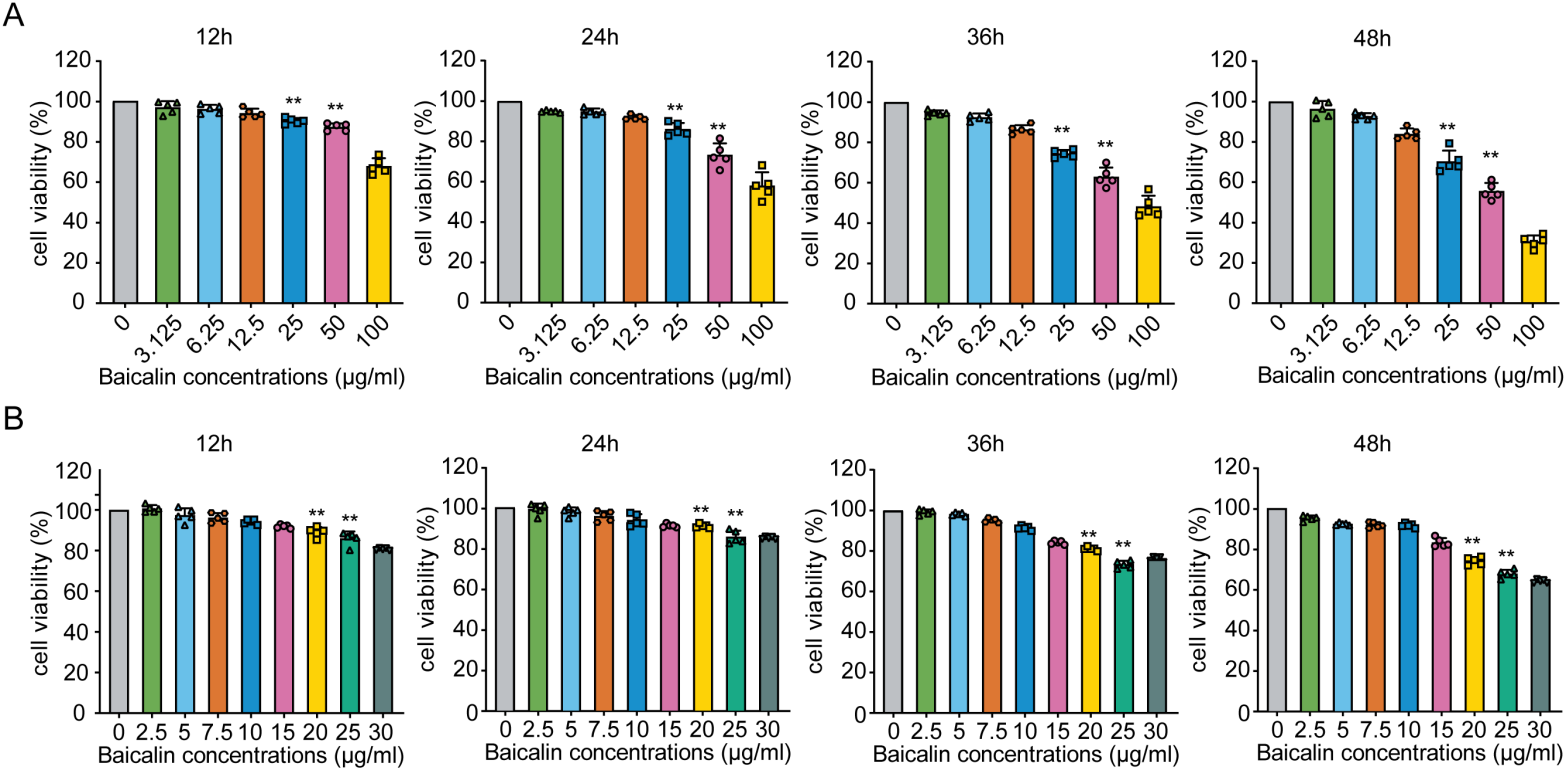
Effects of different concentrations of baicalin on MLE-12 cell viability over time. **(A)** Viability of MLE-12 cells exposed to baicalin concentrations ranging from 0 to 100 μg/mL at 12, 24, 36, and 48 hours post-treatment. **(B)** Screening of baicalin concentrations from 0 to 30 μg/mL. Statistical significance was determined using one-way ANOVA followed by Tukey’s test (**P < 0.01 compared with the control group). Error bars represent standard deviation (SD).

To determine the optimal concentrations of baicalin for antiviral studies, we first assessed the cytotoxicity profile of baicalin in MLE-12 cells over a broad concentration range (0–100 µg/mL). Cell viability remained unaffected (>80%) at concentrations up to 10 µg/mL across all time points (12, 24, 36, and 48 h). However, significant decreases in cell viability were observed at concentrations of ≥25 µg/mL from 12 h onward (**Figure 2A**; *P* < 0.01). Subsequent fine-tuned screening at lower baicalin concentrations (0–30 µg/mL) confirmed that concentrations above 20 µg/mL consistently induced significant cytotoxicity (*P* < 0.01; **Figure 2B**). Importantly, cell viability was maintained at ≥85% at concentrations of 2.5, 5, and 10 µg/mL throughout the 48-hour observation period (**Figure 2B**). Consequently, baicalin concentrations of 5, 10, and 15 µg/mL were selected for further antiviral evaluation, ensuring minimal cytotoxic interference with the interpretation of antiviral activity and immune modulation assays.

### Baicalin alleviates pathological symptoms in mice infected with H9N2 AIV

To evaluate the therapeutic potential of baicalin against H9N2-induced pulmonary pathology, we performed histopathological examinations and calculated lung indices in treated mice. Clinical manifestations were first evident on day 2 post-inoculation in virus-infected mice, including nasal discharge and diminished activity, subsequently progressing to severe respiratory distress by day 6. In contrast, baicalin-treated mice showed notable improvement, characterized by reduced respiratory symptoms, improved activity, and decreased pathological signs compared to the virus-infected model group.

**Figure 3:**
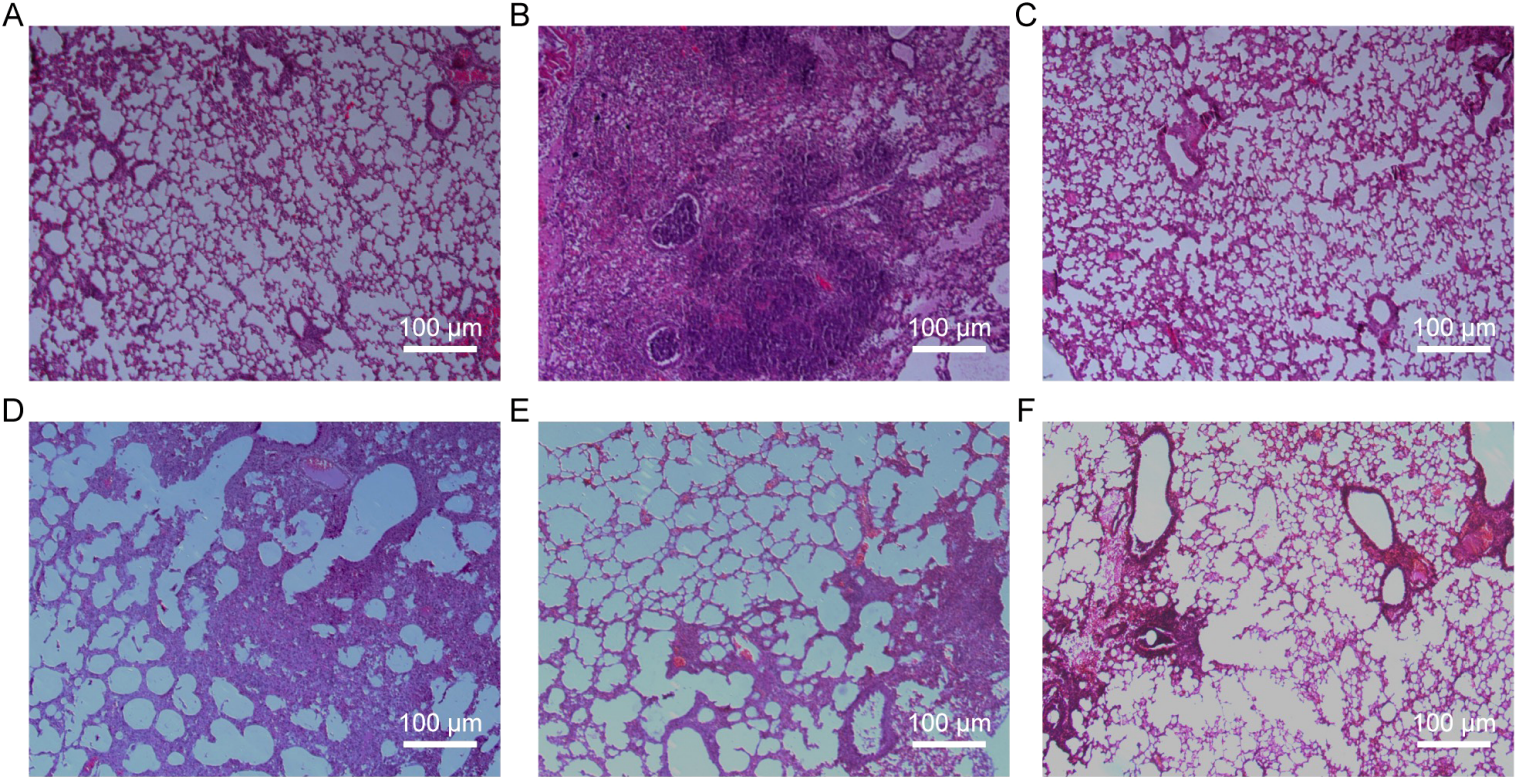
Histopathological evaluation of lung tissues from H9N2-infected mice following baicalin treatment (H&E staining, ×200 magnification). **(A)** Normal group. **(B)** Model group. **(C)** Positive control group. **(D)** Baicalin-treated group (80 mg/kg). **(E)** Baicalin-treated group (160 mg/kg). **(F)** Baicalin-treated group (320 mg/kg). Scale bars = 200 µm.

Histopathological analysis revealed distinct protective effects of baicalin against H9N2-induced lung pathology (**Figure 3**). Lung tissues from uninfected control mice displayed intact alveolar structures, normal bronchial epithelial morphology, and minimal inflammatory cell infiltration (**Figure 3A**). Conversely, mice in the virus model group exhibited severe pathological changes, including widespread lung consolidation, loss of alveolar architecture, extensive inflammatory infiltration, and significant bronchial epithelial damage (**Figure 3B**). Treatment with ribavirin (positive control) markedly alleviated lung injury, retaining relatively normal lung morphology with mild inflammatory infiltration (**Figure 3C**). Importantly, baicalin administration showed dose-dependent protective effects. At the low dose (80 mg/kg), inflammation was moderately attenuated, yet scattered regions with inflammatory infiltration remained visible (**Figure 3D**). Medium (160 mg/kg) and high doses (320 mg/kg) of baicalin provided significant protection, with substantially preserved alveolar structure, reduced inflammatory cell infiltration, and minimal pulmonary congestion (**Figures 3E, F**).

Quantitative lung index analysis further supported these observations, demonstrating significantly lower lung indices in all baicalin-treated groups compared to the virus-infected model group (*P* < 0.01, **Table 2**). These findings collectively indicate that baicalin effectively mitigates H9N2-induced pulmonary inflammation, offering promising therapeutic potential in enhancing respiratory mucosal integrity against influenza infections.

**Table 2:** Effects of baicalin on the pneumonia index of mice infected by H9N2 AIV.

Group	Number	Dose (mg/kg)	Lung Index	Inhibition Rate (%)
Blank Group	10		0.834 ± 0.036	
Virus Group	10		2.982 ± 0.341^##^	
Positive Control Group	10	10	1.135 ± 0.029^**^	61.254
Low Dose Baicalin Group	10	80	1.227 ± 0.091^**^	57.945
Medium Dose Baicalin Group	10	160	1.182 ± 0.15^**^	59.144
High Dose Baicalin Group	10	320	1.076± 0.076^**^	63.073

Compared with the virus group: **P<0.01; Compared with the blank group: ##*P*<0.01.

### ELISA analysis of cytokine profiles in MLE-12 cells and H9N2-infected mice

**Figure 4:**
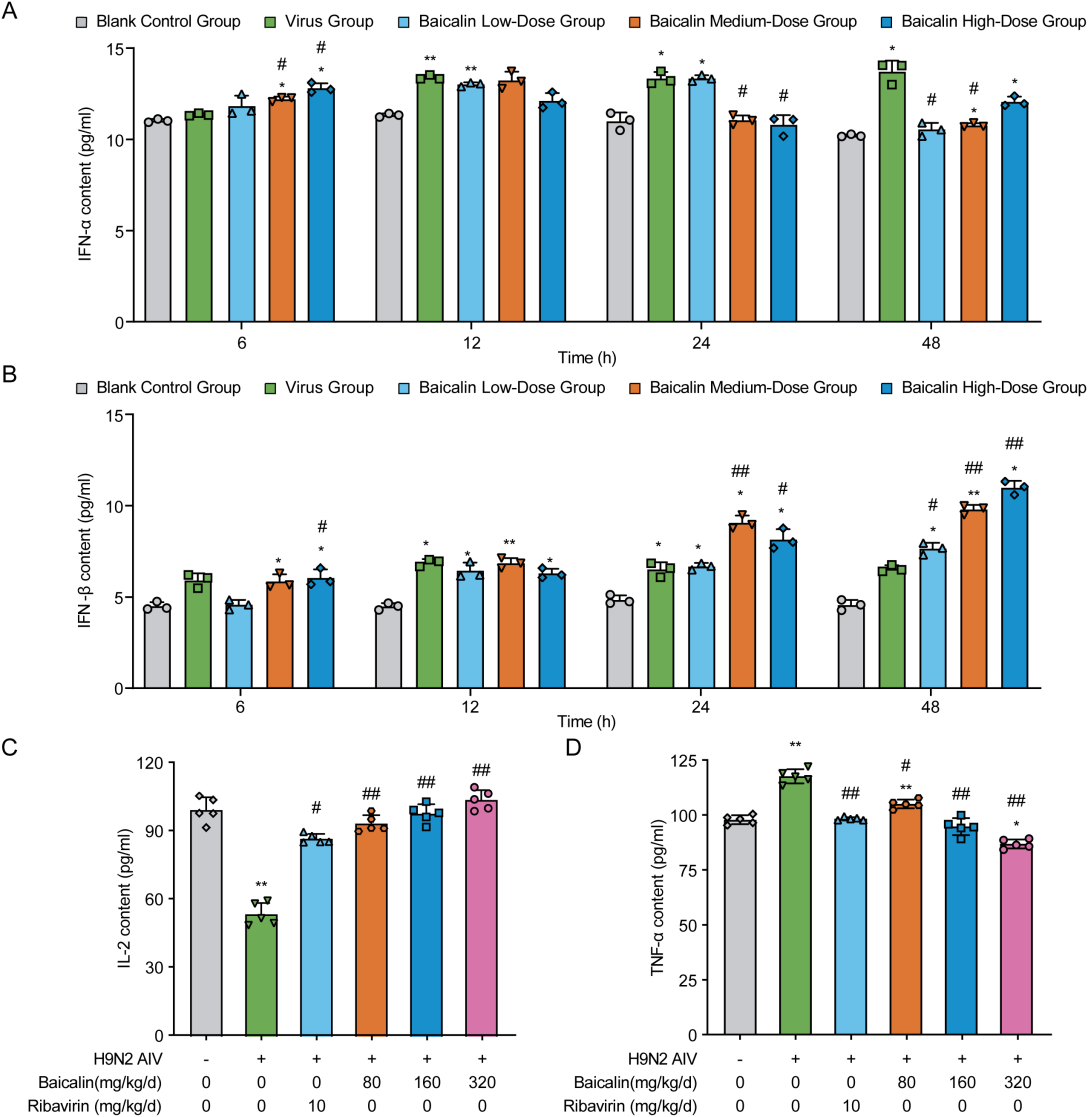
Effects of baicalin on cytokine secretion in H9N2-infected MLE-12 cells and mice. **(A, B)** Cytokine levels in H9N2 AIV-infected MLE-12 cells treated with varying doses of baicalin: **(A)** IFN-α levels and **(B)** IFN-β levels. **(C, D)** Cytokine levels in H9N2 AIV-infected mice treated with varying doses of baicalin: **(C)** IL-2 levels and **(D)** TNF-α levels. Data represent the mean ± S.D. (n = 3) from triplicate experiments. Statistical significance was determined using non-parametric one-way ANOVA. *P < 0.05, **P < 0.01 compared with the control group; #*P* < 0.05, ##*P* < 0.01 compared with the virus group.

We next evaluated the effect of baicalin treatment on cytokine production, focusing on type I interferons (IFN-α and IFN-β) in MLE-12 cells infected with H9N2 AIV. As shown in **Figure 4A**, IFN-α levels significantly increased at 12 hpi in cells treated with baicalin at concentrations of 5 µg/mL (*P* < 0.01). Higher baicalin doses (10 and 15 µg/mL) did not significantly differ from the virus control at 12 hpi, but displayed notable increases at 24 and 48 hpi (*P* < 0.05 and *P* < 0.01, respectively), suggesting a dose- and time-dependent effect on IFN-α induction. IFN-β secretion followed a similar pattern (**Figure 4B**). At 6 hpi, treatment with baicalin at medium and high doses (10 and 15 µg/mL) significantly elevated IFN-β levels compared with the virus-infected control (*P* < 0.05). This trend intensified at 12 hpi, with both medium and high baicalin concentrations significantly increasing IFN-β secretion (*P* < 0.01). By 24 and 48 hpi, IFN-β secretion continued to rise, with the highest dose (15 µg/mL) demonstrating the most pronounced effect (*P* < 0.01 vs. virus control).

*In vivo* cytokine analysis further demonstrated baicalin’s immunomodulatory effects. H9N2-infected mice exhibited significantly reduced serum IL-2 levels and increased TNF-α levels compared with healthy controls (P < 0.01; **Figures 4C, D**). Baicalin treatment reversed these alterations in a dose-dependent manner. Specifically, medium (160 mg/kg) and high-dose (320 mg/kg) baicalin markedly increased IL-2 levels (*P* < 0.01 vs. virus control; **Figure 4C**) and significantly suppressed TNF-α secretion (*P* < 0.01 vs. virus control; **Figure 4D**). Notably, high-dose baicalin displayed stronger cytokine modulation effects than ribavirin, highlighting its potential as an effective antiviral immunomodulator against H9N2 infection.

### Baicalin modulates the expression of antiviral genes in H9N2-infected MLE-12 cells

**Figure 5:**
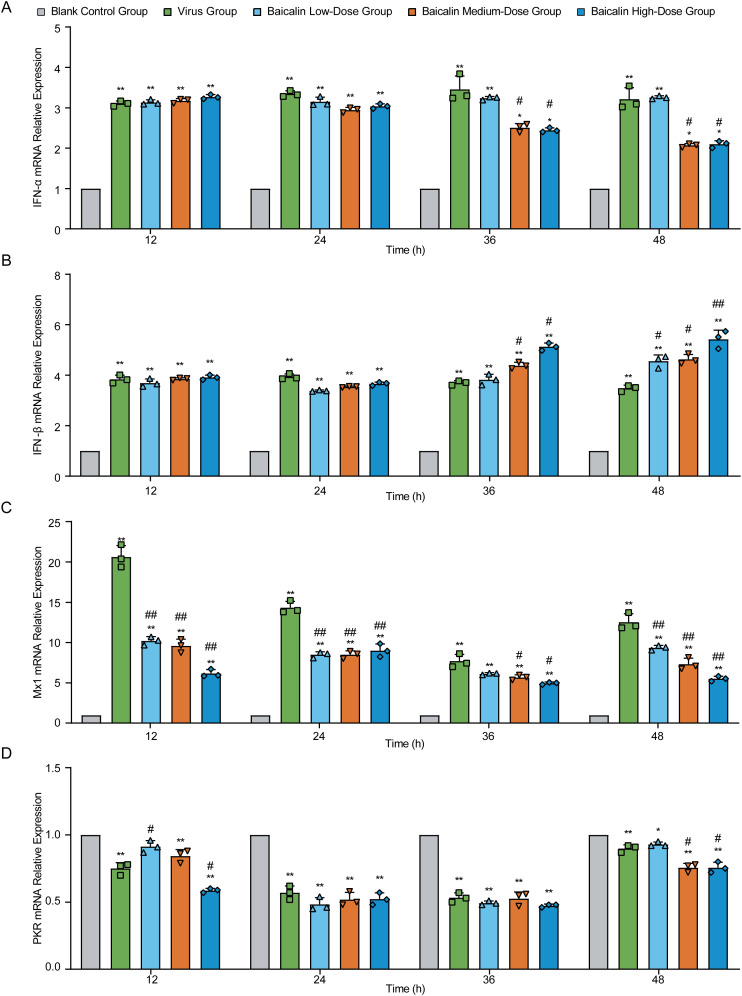
Effect of baicalin treatment on antiviral gene expression in H9N2-infected MLE-12 cells. Relative mRNA expression levels were determined using RT-qPCR at indicated time points (12, 24, 36, and 48 hours post-infection). Genes analyzed include: **(A)** IFN-α, **(B)** IFN-β, **(C)** Mx1, and **(D)** PKR. Treatment conditions comprised Blank Control Group, Virus-Infected Group, and Baicalin-treated groups (Low-dose: 5 µg/mL; Medium-dose: 10 µg/mL; High-dose: 15 µg/mL). Statistical significance was determined using non-parametric one-way ANOVA. *P < 0.05, *P* < 0.01 compared with the control group; #*P* < 0.05, ##*P* < 0.01 compared with the virus group.

To investigate the antiviral mechanisms of baicalin at the molecular level, we measured the mRNA expression of key antiviral genes (IFN-α, IFN-β, Mx1, and PKR) in H9N2-infected MLE-12 cells using RT-qPCR (**Figure 5**). Expression of IFN-α mRNA significantly increased in virus-infected cells compared to controls at all evaluated time points (*P* < 0.01, **Figure 5A**). Baicalin treatment further enhanced IFN-α expression at early time points (12 h), notably at the low-dose (5 µg/mL) concentration (*P* < 0.01 vs. control), whereas higher concentrations (10, 15 µg/mL) slightly reduced this response compared to the virus group (*P* < 0.01). By 36 h, baicalin-treated cells displayed a pronounced increase in IFN-α, with the highest levels observed at 15 µg/mL (*P* < 0.01 vs. virus and control). Elevated expression persisted until 48 h, particularly notable at 10 µg/mL (*P* < 0.05 vs. virus group).

Similarly, IFN-β mRNA was significantly upregulated in the virus-infected group throughout the infection period (*P* < 0.01, **Figure 5B**). Baicalin consistently increased IFN-β expression relative to controls at all concentrations tested, with peak expression at 36 h in the 15 µg/mL group (*P* < 0.01 vs. virus and control). By 48 h, 10 µg/mL baicalin maintained significantly higher IFN-β levels compared to virus-infected cells (*P* < 0.05).

For Mx1, virus infection induced a robust and persistent increase in mRNA levels compared to control cells (*P* < 0.01, **Figure 5C**). Baicalin treatment moderated the virus-induced excessive expression of Mx1, significantly lowering it compared to the virus group at 12, 24, and 48 h (*P* < 0.01). Nonetheless, baicalin-treated groups consistently exhibited significantly higher Mx1 expression compared to controls, suggesting an optimal regulation of antiviral defense. In contrast, PKR mRNA levels were markedly suppressed by virus infection at all examined time points (*P* < 0.01 vs. control, **Figure 5D**). Baicalin further decreased PKR expression at earlier stages (12 h), particularly at medium and high concentrations (*P* < 0.01 vs. control). Over time, PKR expression gradually recovered in all treatment groups, reflecting baicalin’s role in modulating the cellular antiviral response dynamics.

Collectively, these data suggest that baicalin effectively modulates critical antiviral gene expression (IFN-α, IFN-β, Mx1, PKR) in a dose- and time-dependent manner, enhancing antiviral responses while mitigating virus-induced dysregulation, thus highlighting its therapeutic potential against H9N2 infection.

### Baicalin enhances antiviral protein expression in H9N2-infected MLE-12 cells and mice

**Figure 6:**
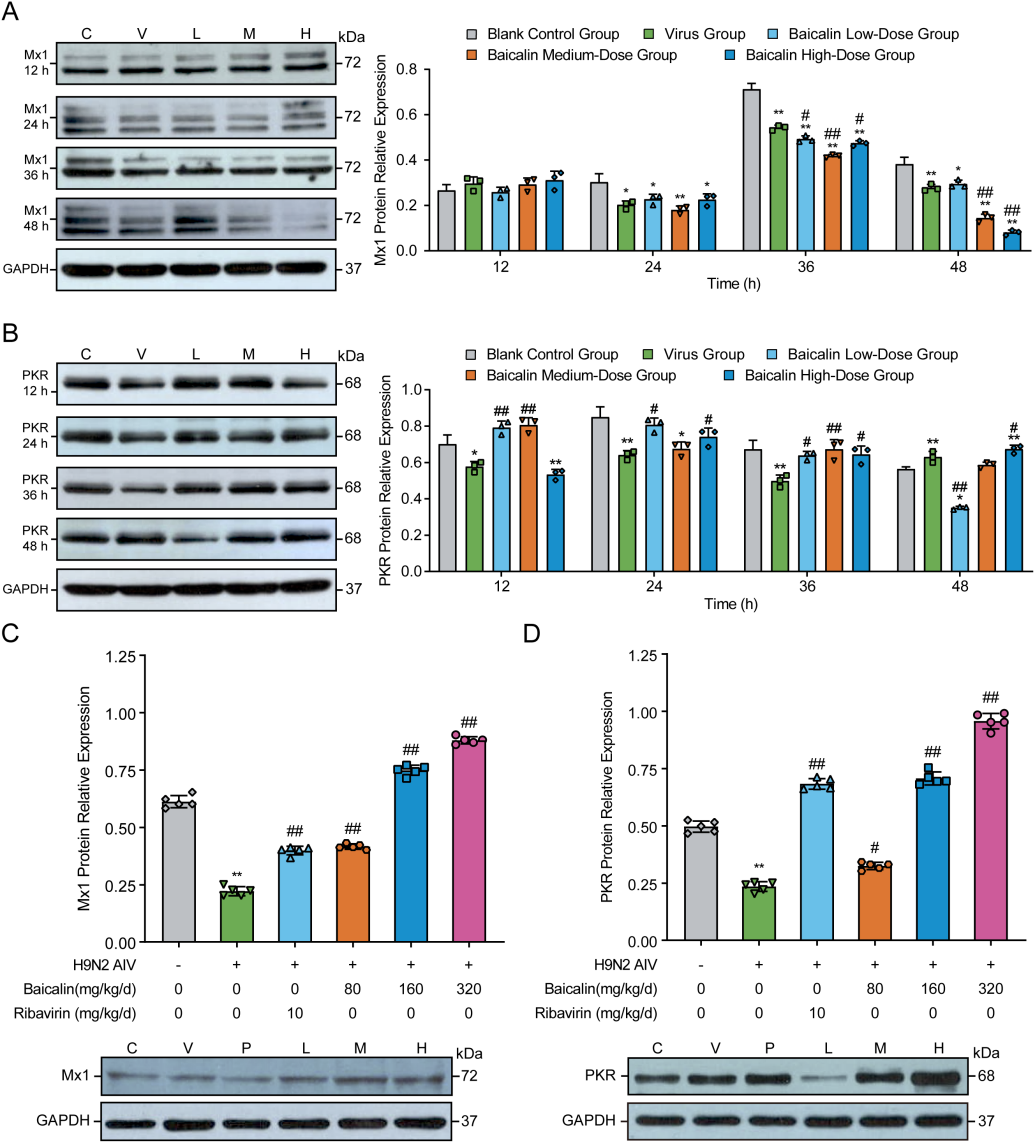
Effects of baicalin treatment on Mx1 and PKR protein expression in H9N2-infected MLE-12 cells and mice. **(A, B)** Western blot analysis and quantification of Mx1 **(A)** and PKR **(B)** protein expression levels in MLE-12 cells treated with baicalin (5, 10, and 15 µg/mL) at 12, 24, 36, and 48 h post-infection. **(C, D)** Protein expression levels of Mx1 **(C)** and PKR **(D)** in lung tissues from H9N2-infected mice administered different doses of baicalin (Low: 80 mg/kg; Medium: 160 mg/kg; High: 320 mg/kg). Ribavirin (10 mg/kg/day) served as a positive control. GAPDH served as the loading control. Statistical significance was determined using non-parametric one-way ANOVA. *P < 0.05, *P* < 0.01 compared with the control group; #*P* < 0.05, ##*P* < 0.01 compared with the virus group.

To further explore the antiviral mechanisms mediated by baicalin, we evaluated the expression of key antiviral proteins, Mx1 and PKR, in H9N2-infected MLE-12 cells and mouse lung tissues using Western blotting. In MLE-12 cells, virus infection alone induced marked increases in Mx1 protein expression at 24 h, peaking at 36 hpi compared with controls (**Figure 6A**; *P* < 0.01). Treatment with baicalin significantly attenuated the virus-induced excessive Mx1 expression in a dose-dependent manner from 24 hpi onwards (*P* < 0.01). The most pronounced reduction was observed in cells treated with 15 µg/mL baicalin at 48 hpi (*P* < 0.01 vs. virus group). Conversely, PKR expression was substantially reduced in virus-infected cells compared to the control at all tested time points (*P* < 0.01, **Figure 6B**). Baicalin treatment restored PKR protein expression in infected cells, with a clear dose-dependent recovery evident at 24, 36, and 48 hpi. Notably, the 15 µg/mL baicalin group exhibited significantly higher PKR levels compared to the virus-infected group at 48 hpi (*P* < 0.01), highlighting baicalin’s potential to restore antiviral defenses compromised by virus infection.

*In vivo*, H9N2-infected mice exhibited significant reductions in pulmonary Mx1 and PKR protein expression compared to controls (**Figures 6C, D**, P < 0.01). Baicalin treatment effectively reversed these reductions, demonstrating significant dose-dependent enhancement of Mx1 and PKR protein levels compared to the virus-infected group (*P* < 0.01). The most robust effects were observed at the high baicalin dose (320 mg/kg), achieving protein expression levels comparable to or exceeding those observed in the ribavirin-positive control group (*P* < 0.01). Collectively, these findings demonstrate that baicalin significantly upregulates critical antiviral effectors (Mx1 and PKR), restoring impaired antiviral responses following H9N2 infection and further underscoring its therapeutic potential as a mucosal antiviral agent.

### Baicalin treatment restores T lymphocyte balance in mice infected with H9N2 AIV

To further explore the immunomodulatory effects of baicalin, we examined the changes in splenic T lymphocyte subpopulations (CD4+ and CD+) and calculated the CD4+/CD8+ T-cell ratio by flow cytometry at days 3 and 6 post-infection (**Table 3**). H9N2-infected mice exhibited significantly reduced CD4+/CD8+ ratios at both 3 and 6 days post-infection compared to uninfected control mice (*P* < 0.01, **Table 3**), indicating severe immune dysregulation induced by the virus.

**Table 3:** Effects of baicalin treatment on the CD4+/CD8+ T-cell ratio in spleen tissues of H9N2-infected mice.

Group	Dose (mg/kg/d)	CD4+/CD8+ T Lymphocyte Subpopulations	
		3d	6d
Blank Group		2.263 ± 0.037	4.566 ± 0.194
Virus-infected Model Group		1.412 ± 0.037##	0.597 ± 0.194##
Positive Control (Ribavirin)	10	2.096 ± 0.054**	3.619 ± 0.142**
Low Dose Baicalin Group	80	1.072 ± 0.034*	2.754 ± 0.116**
Medium Dose Baicalin Group	160	1.836 ± 0.033*	3.571 ± 0.195**
High Dose Baicalin Group	320	1.736 ± 0.073**	4.341 ± 0.108**

Statistical significance was determined using one-way ANOVA with Tukey’s *post hoc* test. Compared with the virus group: **P<0.01; Compared with the blank group: #*P*<0.05 ##*P*<0.01.

Baicalin administration significantly reversed this immune imbalance in a dose-dependent manner. At day 3 post-infection, medium (160 mg/kg) and high (320 mg/kg) doses of baicalin significantly increased the CD4+/CD8+ T cell ratio compared with the virus-infected model group (*P* < 0.01). This restorative effect became even more evident by day 6 post-infection, with all baicalin doses significantly improving the CD4+/CD8+ ratio (*P* < 0.01 vs. virus-infected group). Notably, the high-dose baicalin group demonstrated the greatest restoration, achieving near-normalization of T-cell ratios, comparable to levels in the blank control group.

These results indicate that baicalin specifically counteracts H9N2-induced immunosuppression by restoring the balance between CD4+ and CD8+ T lymphocyte subsets, further highlighting its potential as a valuable immunomodulatory therapeutic for respiratory viral infections.

## Discussion

This study elucidated the dual antiviral and immunomodulatory effects of baicalin against H9N2 AIV using both *in vitro* and *in vivo* models. The findings highlight the ability of this compound to enhance key components of innate and adaptive immune responses, including cytokine upregulation, modulation of antiviral proteins, and restoration of T lymphocyte homeostasis. Baicalin treatment significantly increased the secretion of type I interferons (IFN-α and -β), upregulated antiviral proteins (Mx1 and PKR), and improved the CD4+/CD8+ T-cell ratio in H9N2-infected mice. These results suggest that baicalin not only mitigates viral replication but also counteracts virus-induced immune suppression, providing multifaceted protective effects. Notably, our data reveal a clear time-dependent sequence in baicalin’s immunomodulatory action, characterized by an early induction of type I interferons (IFN-α and -β at 6–12 h), followed by upregulation of downstream antiviral effectors (Mx1 and PKR at 24–48 h). This sequential immune activation aligns with previously reported temporal cascades in innate antiviral responses, wherein initial interferon signaling sets the stage for subsequent expression of antiviral proteins (33, 34).

Type I interferons are pivotal mediators of antiviral immunity, particularly against influenza virus infection (35). IFN-α and -β activate the JAK/STAT pathway, inducing interferon-stimulated genes such as Mx1 and PKR, critical for controlling viral replication (36, 37). The observed temporal pattern of IFN-α/β production—characterized by rapid induction at 6–12 hours followed by sustained elevation at later time points (24–48 hours)—suggests baicalin enhances both initial pathogen recognition and sustained antiviral signaling, distinguishing it from single-target antivirals. Previous studies have similarly demonstrated that flavonoids, including baicalin, promote interferon production by modulating toll-like receptor and NF-κB signaling pathways (36, 38). Our *in vitro* results demonstrated complex, dose- and time-dependent changes in cytokine secretion, where lower concentrations of baicalin transiently suppressed IFN-β at early time points (6 h), while medium and high doses significantly enhanced cytokine levels at later time points (24–48 h). This complexity might reflect baicalin’s dual action: initially dampening excessive inflammatory responses, possibly through negative feedback regulators such as SOCS1 (39), then promoting sustained antiviral immunity at later stages and higher doses.

The observed upregulation of Mx1 and PKR provides further mechanistic insights into baicalin’s antiviral activity. Mx1, an interferon-stimulated gene, inhibits influenza virus replication by targeting viral ribonucleoproteins and interfering with their nuclear import (40); similarly, PKR suppresses viral protein synthesis and triggers apoptosis in infected cells, effectively limiting viral spread (41). Interestingly, our *in vitro* results revealed a discrepancy in Mx1 expression: although viral infection significantly elevated Mx1 mRNA levels, it unexpectedly reduced corresponding protein levels. Baicalin treatment restored this imbalance, possibly by counteracting viral NS1 protein-mediated translation inhibition (42, 43) or stabilizing Mx1 protein through post-translational mechanisms, which merit further investigation. These complex *in vitro* dynamics contrast with more straightforward *in vivo* findings, where baicalin consistently enhanced protein expression levels of both Mx1 and PKR, clearly reflecting the compound’s potential for stabilizing antiviral defenses within the more integrated immune environment of the intact organism. Our results suggest that baicalin not only enhances antiviral protein expression but also regulates their dynamic expression to prevent excessive immune activation, a balance that is crucial in limiting influenza-induced immunopathology (44, 45). The dose-dependent divergence in PKR expression, especially evident *in vitro*, may represent baicalin’s ability to finely balance between promoting antiviral activity and preventing excessive cellular apoptosis, thereby limiting tissue damage—a mechanism that appears to be efficiently orchestrated *in vivo* under physiological conditions.

The restoration of the CD4+/CD8+ T cell ratio in baicalin-treated mice highlights its immunomodulatory effects on adaptive immunity. Viral infections, including H9N2, are known to disrupt T cell homeostasis, leading to immunosuppression (46, 47). Baicalin’s preferential restoration of CD4+ T-cell levels, without concurrent overstimulation of CD8+ populations (**Table 3**), suggests that it functions primarily to rebalance T-cell subsets rather than actively selecting specific cell types. This rebalancing may reduce the risk of immunopathology by avoiding excessive immune activation (48). Similar modulatory effects on T-cell subsets have been documented previously; for example, baicalin restored CD4+/CD8+ ratios disrupted by respiratory syncytial virus infections (49). Other flavonoids, such as quercetin and epigallocatechin gallate, also modulate T-cell responses in influenza models, further supporting flavonoids as broadly applicable agents in antiviral immunotherapy (50, 51). Our current study primarily focuses on adaptive T-cell dynamics; however, recent literature has hypothesized potential flavonoid-induced “trained immunity”—an innate immune memory characterized by enhanced nonspecific responses upon pathogen re-exposure mediated by cells like macrophages and NK cells (52). Although our data do not directly address this phenomenon, it represents an intriguing future direction that warrants further investigation, particularly regarding whether baicalin influences epigenetic reprogramming associated with trained immunity to provide broader antiviral protection.

From an applied perspective, these findings have significant implications for the development of alternative antiviral therapies. Influenza viruses, particularly emerging strains such as H9N2, pose a persistent challenge because of their capacity for antigenic drift and vaccine evasion (53). Notably, previous studies have demonstrated that baicalin exhibits antiviral activity against multiple influenza virus subtypes, including H1N1 and H3N2, suggesting it possesses broad-spectrum antiviral potential (16). The dual role of baicalin in modulating innate and adaptive immunity positions it as a promising candidate for combination therapies aimed at reducing disease severity, enhancing vaccine efficacy, and mitigating the risk of resistance associated with conventional antiviral drugs. Moreover, its natural origin and established safety profile support its potential as a complementary therapy for managing viral infections in both human and veterinary contexts (54). However, while these findings provide strong preclinical evidence, further investigations are needed to translate these results into clinical applications.

Despite the promising findings, this study had several limitations that warrant further investigation. First, while the murine model used in this study is valuable for investigating host-pathogen interactions and immune responses, it does not fully recapitulate influenza infection in poultry or humans. The absence of avian-specific factors like tracheal cilia dynamics and air sac anatomy may limit direct extrapolation to poultry. Future studies using avian models or primary avian cells would enhance the translational relevance of our findings. Second, a major gap in our study is the lack of pharmacokinetic and toxicity assessments. While baicalin has demonstrated a favorable safety profile in previous studies, detailed investigations into its bioavailability, metabolism, and potential off-target effects are essential for its clinical development. Third, the antiviral effects described here are specific to H9N2 AIV. Future work should determine whether baicalin demonstrates similar efficacy against other influenza subtypes or unrelated viruses.

In summary, this study provides compelling evidence that baicalin modulates both innate and adaptive immune responses, enhancing host defense against H9N2 infection. These findings support the potential of this compound as a natural therapeutic agent, offering a foundation for future investigations into its application in combating influenza and other viral diseases.

## Data Availability

The raw data supporting the conclusions of this article will be made available by the authors, without undue reservation.
